# The Infomóvel—An information system for managing HIV/AIDS patients in rural areas of Mozambique

**DOI:** 10.1186/s12911-023-02281-6

**Published:** 2023-09-18

**Authors:** E. Karajeanes, D. Bila, M. Luis, M. Tovela, C. Anjos, N. Ramanlal, P. Vaz, L. V. Lapão

**Affiliations:** 1 Fundação Ariel Glaser Contra O SIDA Pediátrico, Avenida Agostinho Neto N° 620, Maputo, Mozambique; 2 Global Health and Tropical Medicine, Instituto de Higiene E Medicina Tropical, Universidade Nova de Lisboa, Rua da Junqueira, N° 100, 1349-008 Lisboa, Portugal

**Keywords:** mHealth, Design science research methodology, Community health workers, HIV, ART, Mozambique

## Abstract

**Background:**

Mobile health is gradually revolutionizing the way medical care is delivered worldwide. In Mozambique, a country with a high human immunodeficiency virus prevalence, where antiretroviral treatment coverage is 77% accompanied by a 67% of retention rate, the use of mobile health technology may boost the antiretroviral treatment, by delivering care beyond health facilities and reaching underrepresented groups. Leveraging new technologies is crucial to reach the 95–95-95 United Nations target by 2030. The design, development, implementation, and evaluation of a mobile health platform called Infomóvel were covered in this article. Its intended use involves collaboration with community health workers and aims to increase human immunodeficiency virus patient access, adherence, and retention to care.

**Methods:**

Using the Design Science Research Methodology, Infomóvel was created, as well as this publication. The explanation of various actions includes everything from problem description to observational study and goal-following for a solution, which results in the design and development of a platform proposal. Before the utility assessment of Infomóvel was conducted to make adjustments, a demonstration phase was conducted in one region of Mozambique.

**Results:**

The initial subjects of the Infomóvel flowchart and physical process design were patients receiving antiretroviral medication who were enrolled in the patients tracking system and who had consented to home visits. The case manager examines the file before importing it into the Infomóvel database stored on a cloud server using the website www.commcarehq.org. The case manager application synchronises with the Infomóvel server database, enabling the import of latest data and access to the lists of new patients and community health workers. The community health worker uses his phone to access his application, which allows him to record the geographic coordinates and sort the list of patients by priority and type of visit.

**Conclusion:**

Results from Infomóvel add to the growing body of data showing that mobile health techniques are beneficial for managing stable individuals with chronic conditions in Mozambique. These approaches can be scaled up and better utilised. However, additional studies should be conducted to quantify the resources needed to implement on a larger scale.

## Background

Mobile health (mHealth) solutions can be advantageous because of their adaptability and lower costs in addressing organizational challenges in the healthcare sector, such as dealing with a lack of health professionals, enhancing health surveillance services, and low- and middle-income countries. “(LMIC)” [1].

It is recognized that managing human immunodeficiency virus (HIV) epidemics presents a serious challenge in providing access to and in managing health information at the primary and community level, in most “LMIC”, including Mozambique [2, 3]. According to the Joint United Nations Programme on HIV/AIDS (UNAIDS) 2019 report, Mozambique has one of the highest HIV prevalence rates in sub-Saharan Africa with over 2.2 million people living with HIV there.. The same report states that only 72% of People Living with HIV (PLWHIV) are aware of their status and, of those, only 77% are on antiretroviral treatment (ART) [4].

By 2030, the UNAIDS 95–95-95 initiative targets are 95% of HIV-positive people aware of their status, 95% of those who are aware of their status to be on ART, and 95% of those on ART to achieve virological suppression. This necessitates innovative service delivery models being developed by programmes globally, including those in Africa, to include all HIV-positive people in the care cascade and close the current gap [4]. To achieve the government’s goal of treating nearly one million HIV-infected people by 2017, the Ministry of Health (MoH) has displayed commitment to community-based approaches to care and treatment. One such model includes the Community Adherence and Support Groups (CASG), a strategy that groups patients together in order to establish rotational drug collection and distribution system in the community [5]. Other community-based approaches are also promising, such as home visits by community health workers (CHWs) and community-based counselling, which have shown gains in identifying high-risk patients (family members of index patients) and a decrease in stigma in the community [6]. Following MoH recommendations, Ariel (a national NGO) adopted the “index case model” to provide (HIV) and tuberculosis (TB) services in the community. Through this approach, cohabiting relatives of the index patient are screened for HIV and TB, where follow-up visits upon treatment initiation are made, and active search of default patients is performed. The use of community-based health professionals to reach, test, and refer HIV and TB patients has received a great deal of support in literature and experience from sources outside of Mozambique [7–12].

While Mozambique is making every effort to increase access to HIV care, the MoH reported in 2019 the retention rate—the percentage of patients on ART who are still alive and taking their medication one year after beginning ART—was 67% nationally and 74% and 63% in the provinces of Maputo and Cabo Delgado, respectively [13, 14]. Lack of resources and travel time to medical facilities are just two of the many issues that pose serious obstacles to patients' ability to adhere to HIV and TB therapy and remain on it.

Complementing the community-based approach, mobile telephone technology is emerging as a tool to support chronic disease management via Internet [15, 16]. Mobile phone ownership and use is experiencing exponential growth in Africa [17]. These two factors have led to the recent rise in research efforts regarding the use of mobile phones to enhance the access to healthcare services like HIV and TB care. Several studies demonstrated the positive impacts of using mobile technology to strengthen community-level management of suspected HIV and TB cases. There are positive impacts also on reinforcing links between CHWs and district health facilities, on improving voluntary counseling and testing, linkage to care, and treatment adherence [15, 18]. Additional regional evidence supports that community-based mHealth approaches improved social support, privacy (contrary to expectations), and reduced stigma because of community-based patient tracing and counseling, however no changes detected in rates of virologic failure, adherence, mortality, or retention, except virologic failure in the long term (> 96 weeks) [19–21]. The use of mHealth to improve ART adherence has a relevant evidence base [22] and the strongest evidence base were find in sveral descriptive [23, 24] and quasi-experimental studies [24–26] conclude that text message reminders are acceptable, feasible, and useful for improved treatment adherence among people living with HIV in resource-constrained settings. The usefulness of mHealth for adherence to ART was established by two impact studies, both were RCTs (Randomized controlled trials), carried out in Sub-Saharan Africa. During a 48-week intervention period in Kenya, Pop-Eleches et al. discovered that treatment adherence significantly increased among patients who were randomly assigned to receive weekly text message reminders [27]. The authors concluded that short weekly reminders were significantly more effective than daily text reminders. Consistent with these findings is another Kenya-based RCT, Lester et al. they revealed that patients receiving weekly SMS reminder messages had significantly higher rates of viral suppression and ART adherence compared to the control population [28].

Furthermore, increasing amounts of data are available to support the implementation of mHealth strategies in places like Mozambique [29]. In the Maputo province's three public health institutions from 2011 to 2012, SMSaude, a randomised control trial, compared routine care to regular text message reminders for HIV-infected patients on ART. Among urban patients, text messages significantly improved retention in HIV care and newly initiated patients on ART (< 3-months) had lower attrition compared to control patients, especially urban patients [22].

The body of research on the economics of mHealth programmes is generally expanding, but few studies have taken into account the costs of community-based mHealth initiatives to reach, test, and refer HIV and TB patients. According to a recent South African study, active home HIV testing and counselling with high coverage (door-to-door) is very cost-effective (less than 1/6 GDP per DAY saved) [29]. According to Nascimento et al., by May 2014, the cost per person searched for and returned to the health institution using mobile phones was $36.51, whereas the cost per person searched for and returned to the health facility was $35.57. This endeavour could be seen as cost-saving when compared to the direct cost of the laboratory tests to start a patient on medication in Mozambique, which is $40 USD [30].

The purpose of this intervention is to create and assess the usefulness of a mobile health platform ("Infomóvel") that would improve the relationship between the community and medical facilities, it also permits the systematic follow-up of HIV-positive patients on ART in the community, and additionally to enhance the detection of HIV-positive patients from index cases using the Design Science Research Methodology (DSRM).

## Methods

The DSRM was used to design, develop, implement, and evaluate an information system. DSRM has been successfully applied in health, on development of warehousing solution data to support necessary analysis in public health policy [31], on implementation of online services for pharmacies to manage chronic diseases [32] and also in use of an innovative surveillance system on the use of antibiotics in order to support clinical decision making [5].

First, we described and validated the issue that needed to be addressed using a 2016 observational study. Following that, the following objectives for a solution were defined, leading to the design and development of a platform proposal DSRM starting in 2016: Infomobile: the Infomóvel. After the design and development, demonstration phase was carried out in one region of Mozambique, before the utility evaluation of the web platform was carried out to adjust on the platform to meet the expectations of the main stakeholders in the process. The final step was disseminating the findings, and this report will be the primary product along with data sharing with MOH (Table 1).

**Table 1:** Steps of Infomóvel design, development, implementation

Step 1 Month 1 – 3	Identify Problem (2016) Problem conceptualization and validation, and design of program theory and stakeholder’s engagement (objectives of the solution)
Step 2 Month 4 – 7	Defining the objectives of a solution (2016) Prototype design and presentation to stakeholders
Step 3 Month 8- 10	Design and platform development (August to October 2016)
Step 4 Month 8- 12 Month 12–24	First Pilot phase—Prototype testing on the field in Cabo Delgado, Montepuez District (August to December 2016) Second Pilot Phase: Adjustments to platform and pilot of the HIV Module (January 2017 to September 2019)
Step 5	Evaluation of the utility of web platform (mid-term evaluation) Occurred concomitantly as the pilot, includes (resulting from evaluation): • Development and incorporation of TB and PMTCT mother-baby pair modules • Concomitant piloting of TB and PMTCT mother-baby pair modules
Step 6	Dissemination of the results • Share preliminary results and platform with other implementing partners

While defining the solution, key assumptions were made: (1) The mobile network company had to have good coverage in rural communities in the targeted provinces, (2) platform to be developed had to be user friendly since CHWs have limited technological knowledge and (3) cultural habits should be considered in all steps of development and implementation.

The implementation using the DSRM methodology was performed in six phases as shown in Table 1, and described as follows:

### Step 1- Identifying the problem

To improve access and treatment retention, active search, program was rolled out to complement the facility care for people living with HIV. When patients in treatment fail to return at appropriate time for consultations or pick-up of medication, health facilities deployed community workers to locate those patients, counsel them and assist them in returning to the facility to recommence treatment. Patients were considered defaulters on their ART medication if they fail to collect their medicine from between 0 to 59 days, after which they were considered as lost-to-follow-up.

The active paper-based search system remains an ad-hoc system throughout the country, with different processes and procedures depending on the health facility and region. In this process several stakeholders were involved such as the Ministry of Health, donors and MoH implementation partners, including Ariel and the University. The active search system remains an ad-hoc system throughout the country, with different processes and procedures depending on the health facility. Therefore, outcomes of interventions were less consistent and less robust.

It was obvious that there weren't enough CHWs to meet the need for HIV services, and that there was a need to coordinate the various CHWs' operations, which couldn't be done with a paper-based implementation. A decision was made to create and construct a mobile application that will assist, need SOP to be followed step-by-step, produce accurate real-time data, and also enable linking to the electronic patient tracking database at the healthcare facilities.

### Step 2- Defining the objectives of a solution

This step provides the functionalities required for the solution to properly address the problem. To evaluate the effectiveness of the link between the community and the health facility (HF) upon referral and to produce timely, accurate data for programmatic and surveillance purposes, it was necessary to ensure intervention uniformity, quality, and integrity among CHWs.

As a result, in our sample health facilities, a paper-based index case methodology was adopted for 6 months prior to the implementation of the Infomóvel platform in order to familiarise the health facility and the Community health workers with the index case methodology. Index case approach is the practice of finding other high-risk individuals, in this example those residing in the household of the index patient, by first identifying the index case, who is a proven HIV-positive or TB patient. The paper-based index case programme actively looked for HIV-positive and TB patients, including those who were also co-infected with HIV and TB, who had missed appointments, lab pick-ups, or other required visits to the medical facility (actively in treatment default). Newly identified community members with HIV and TB who were referred to, showed up to, and registered at the medical facility can also be considered index patients.

### Step 3-Design and web platform development

The Infomóvel application was developed using DSRM [5, 31, 32] from August to October 2016. The primary stakeholders were involved, and a stronger connection to the working processes was made using the co-design methodology. The issue was initially conceptualised, and potential inputs needed to implement the programme were assessed (i.e., the objectives of the solution). Once local leaders and government officials agreed, Ariel teamed up with Dimagi, the owner of CommCare (an open-source mobile data gathering platform), to assist in putting the solution in place that was appropriate for the situation.

The program for HIV counseling and testing in the community based on the index patient, lead Ariel to designing and developing a new strategy named “Infomóvel” which aimed to ensure effective and efficient linkages between the community and the health facilities, through automatic referral of patients from community. The Rural Hospital of Montepuez was selected and the APE (“from Portuguese, Agente Polivante Elementar”, basic community agents) network linked to this area were involved. The health personnel assigned to this health facility participated in the survey and systematization of information to model the information system while the staff of the District Directorate of Health, Women and Social Welfare of Montepuez and the Provincial Directorate of Health of Cabo Delgado participated in the definition of strategic protocols/agreements for the implementation of the platform.

The construction of the Infomóvel Platform was based on an open-source technology (Open Source), namely CommCare, located at https://www.commcarehq.org, developed in 2007. This technology, which enables the creation of mobile applications based on phones and tablets and is extensively utilized in more than 50 countries, is technically sophisticated and allows for the recording of evidence or facts via mobile devices in low-cost settings (www.dimagi.com). The web application runs over Android platforms, and it is supported by a secure database located at Ariel facilities.

This platform allows registration of home visits, testing, counseling, cohabiting family and referrals.

**Fig. 1:**
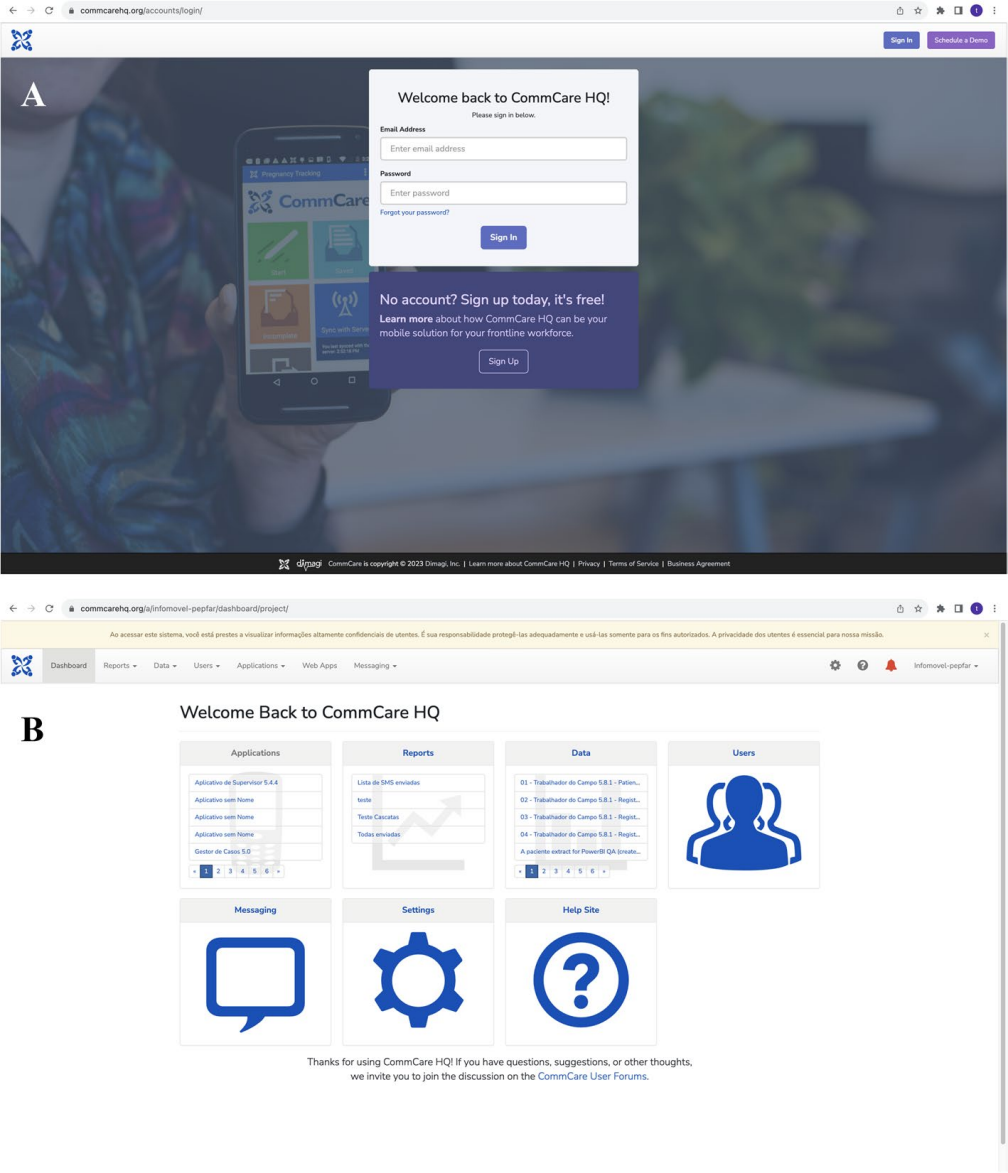
Commcare web app

To access the Commcare web App, one has to sign up as seen in Fig. 1A with a registered email by imputing your email address and password as an old user and signing up as a new user. Upon signing/logging in it will open as seen in Fig. 1B with features like Applications, Report, Data, users, messaging and help site.

### Step 4- Pilot phases

First pilot phase: From August through December 2016, the Montepuez district of Cabo Delgado Province hosted a demonstration of the Infomóvel application. 22 CHWs and about 244 HIV patients took part in this demonstration. The necessity to test the platform in various environments was underscored by the good findings of the data flow testing in Montepuez.

Second pilot phase: From January 2017 to September 2019, the Infomóvel platform's HIV module was modified and tested at five additional health facilities in the Maputo province. APE were replaced with CHWs during this second experimental phase, with higher educational levels and smartphone handling skills.

### Step 5- Evaluation of the utility of web platform

Data analysis was performed using excel data from Infomóvel implementation, demonstration, and evaluation in one health facility in Cabo Delgado and in the five health centers in Maputo province.

### Step 6- Dissemination of the results

During the research project, the findings were disseminated via oral and written reports. All participating health personnels in the Infomóvel pilot phase, as well as the district and provincial health directorates and other partners, were informed of the results of the pilot, which were also presented at an international conference. The report included detailed data collection procedures, data summaries, data analysis and a summary of key findings. Later, the platform and strategy were expanded to other geographical areas in the country by other clinical implementing partners.

## Results

### Infomóvel information system

**Fig. 2:**
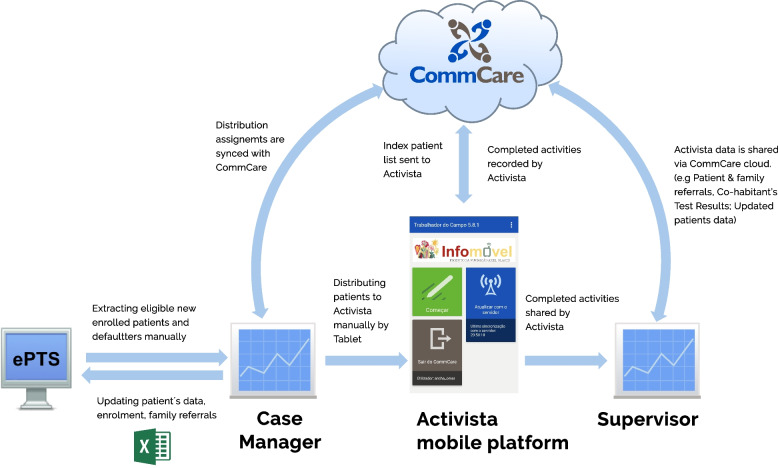
Infomóvel dataflow framework

The ART patients who have accepted home visits and are registered in patients tracking systems (ePTS or OpenMRS) are the first to be included in the Infomóvel flowchart and physical process architecture. The case manager verifies the file's compliance before importing it into Infomóvel’s database using the website www.commcarehq.org, which is located on a cloud server (Fig. 2).

Synchronization with the Infomóvel Server database is performed in the case manager application, which can be accessed by Tablet or PC. This allows the program to reflect recently imported data. The list of CHWs and the list of new patients are currently available to the case manager. It assigns the patients to the CHWs based on their residential proximity. The case manager has in the application, the function of checking data congruence before allocation and making correction. The list of patients assigned to him can be accessed by the CHW via his phone and his application. After synchronizing with the Infomóvel server database, CHWs can update the list. The CHWs application shows the list of patients according to the visit priority to be made according to different patient profiles such as HIV positive pregnant and lactating women, HIV exposed child, child and adult ART patients, co-infected HIV/TB patients and TB patients. The platform provides three types of visits, namely the First Patient visit, Follow-up visit and lost-to-follow up visit. The application, which is kept at the home of the community health worker, stores the exact location, and all the data that the worker gathers, including demographics, diagnoses of various symptoms, estimates of ART adherence, records of cohabitants and their results of rapid tests, counseling, health education, and referrals of patients to medical facilities. The index case may also be referred for an HIV + test if the cohabitants exhibit any of the predicted symptoms, have low ART adherence, or are actively being followed up on.

**Fig. 3:**
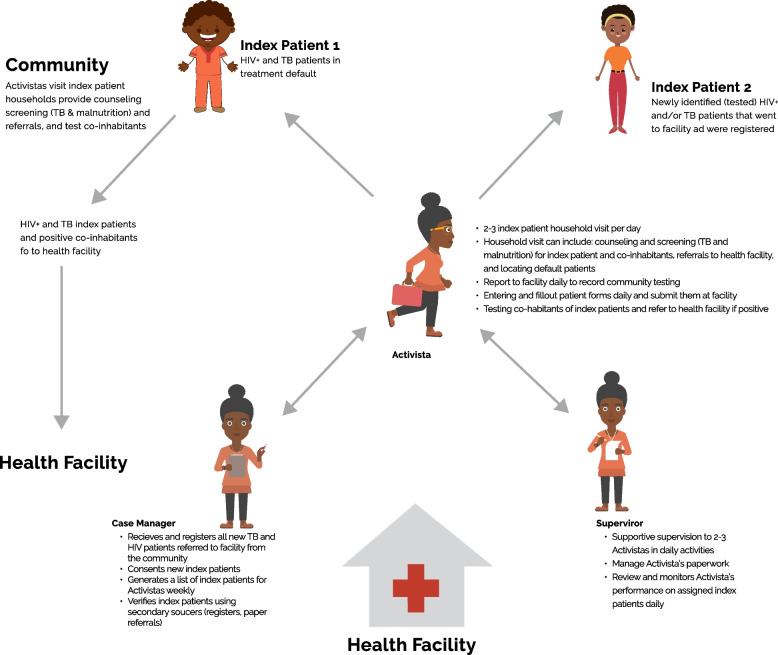
Paper-based index case program process

**Fig. 4:**
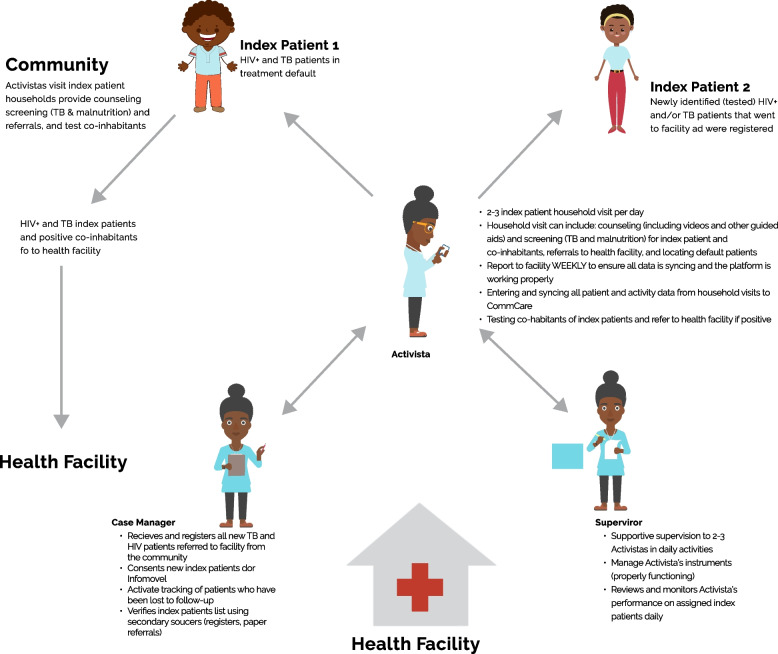
Infomóvel program functionalities

Ariel improved the active search method by first adopting an index case program on paper (Fig. 3), and then gradually phasing in the mobile-based Infomóvel platform, with the index case approach digitized and additional HIV counseling tools (Fig. 4).

The proposed solution is the outcome of a program theory design that includes workflow, what must happen at the HF (health workers, case managers, and patient flow), what must happen at the community level (CHWs, and reference materials), and what must happen in terms of clearly defined desired outcomes. The defined objectives were to increase the identification of HIV positive individuals, increase the linkage to HIV services, and increase the adherence to treatment and retention into care as presented in Fig. 3.

### Paciente management process using the Infomóvel information system

#### First Setp: Paper-based index case program

At the health facility, a list of default patients was generated weekly by a case manager, and CHWs were deployed to locate those patients. CHWs made an initial household visit to each assigned index patient, to provide counseling and assist them in going to the facility for treatment with a referral slip. Household visits to each index case were conducted within one week of being assigned to a *community health worker* (basic community agent), and subsequent visits to the household at 30, 90, and 180 days. All enrolled index patients were to be followed for 6 months using paper-based methodology regardless of when they enrolled. These household visits serve as opportunity for counseling and HIV testing for co-inhabitants, checking for treatment adherence, screening for TB and malnutrition and follow up of mother baby pair enrollment on the Prevention of Mother to Child Transmission (PMTCT) program. After 6 months, index patients were referred to community supporting groups, the *Grupos de Apoio a Adesão Comunitária* (GAACs) model, is a strategy that groups patients together in order to establish rotational drug collection and distribution system in the community. To create the patient list and assign patients to community health workers in their catchment areas, the case manager collaborated with the health facility's data entry manager. This list, which is replicated and sent to the CHWs at the healthcare institution, contains the patient's name, address, contact information, and the reason for the search (active default or new patient). The index patient list was triangulated with data from patient file, pharmacy records, and electronic Patient Tracking System (ePTS) to ensure accuracy. Ariel was in charge of organizing and offering technical support for the paper-based index case program and also implemented the approach by overseeing the cadre of CHWs and handling the cascade data from community testing, consultations, and results. The number of index patients that CHWs contacted, as well as information about the home community and co-occupants, are all reported at the end of each month. All of these features must be supported by the digital platform, which must also be replicated in a web application. This prototype for HIV services was presented to the stakeholders, later the first prototype of the mobile application “Infomóvel” was built with the HIV module (August to October 2016).

#### Second step: The mobile-based Infomóvel platform

The Infomóvel application was developed using DSRM [5, 31, 32] from August to October 2016.

The co-design methodology was utilized to involve the key players and more closely connect it to the working procedures. The problem was initially conceptualized, and potential inputs needed to implement the program were assessed (i.e., the objectives of the solution). Ariel teamed up with Dimagi, the owner of CommCare (an open-source mobile data gathering platform), after local leaders and government officials had expressed interest in the idea, to assist in putting the solution in place that was appropriate for the situation.

In Mozambique, active search approach with paper-based forms typically requires CHWs to travel long distances to the health facility to retrieve the list of patients in their community, return to their communities to search for the patient, and then once again return back to the health facility to complete the search [33]. To streamline the active search process, Infomóvel introduced a mobile web application that allowed CHWs to receive their patient information via their mobile device and send updates and progress back to the case manager and supervisor located at the health facility. Due to the need for reading and writing skills in order to be able to administer HIV tests, CHWs were the only cadre eligible to take part in the Infomóvel pilot. By strengthening the connections between CHWs and district health facility clinicians, the Infomóvel platform was developed to improve patient access, tracking for HIV + and TB patients, and retention of patients with HIV and co-infected HIV/TB patients (Fig. 4). The platform guides CHWs to provide counselling for default ART patients, index patient with low adherence to ART, disclosure of HIV status between partners, discordant couples, pos-tested counseling for HIV positive identifies patients and customized messages and job aids (videos and tutorials) depending on a series of scenarios as well as provides access to support videos in several local languages as well. Ariel provided smartphones and training to ensure that timely referrals were made and to forge strong communication links between CHWs and district health facility case managers for technical advice, follow-up of registered patients and tracking of patients who had been lost to follow-up, as well as cases of suspected co – infected HIV/TB and HIV.

### Service modelling

**Fig. 5:**
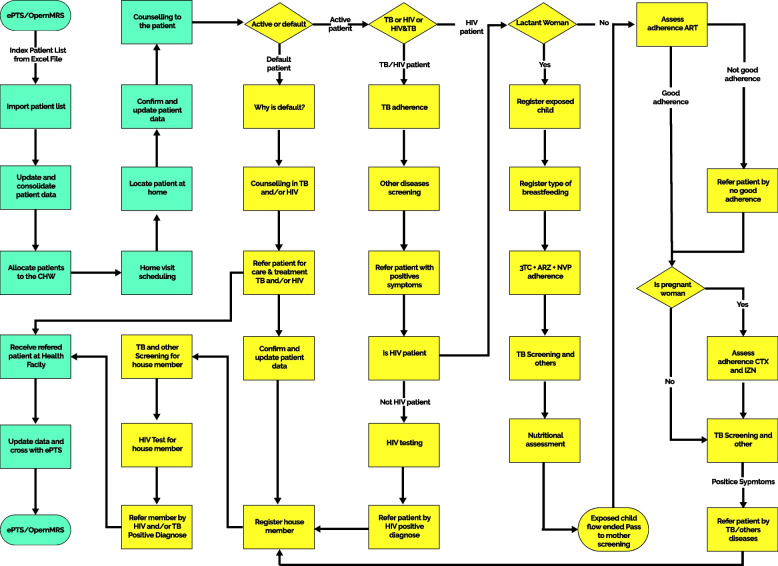
Infomóvel flowchart processes

The modelling phase at the end of step 2 produced an initial plan for community follow-up of HIV-positive patients through an mHealth system (Infomóvel). The prosecution of all DSRM CHWs allowed the development of a new project that focused on the architecture of an HIV-positive community follow-up system based on HIV program management. The prototype shown in Fig. 5 depicts the logical steps taken by the Infomóvel platform, including its flow and all of the services offered there. This prototype also specifies the locations and time at which clients interact with the services in hospitals and communities.

#### Evaluation

**Fig. 6:**
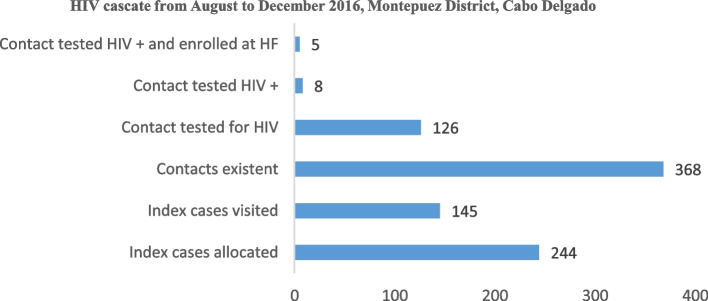
Infomóvel first pilot phase testing results from august to December 2016, Montepuez District, Cabo Delgado

**Fig. 7:**
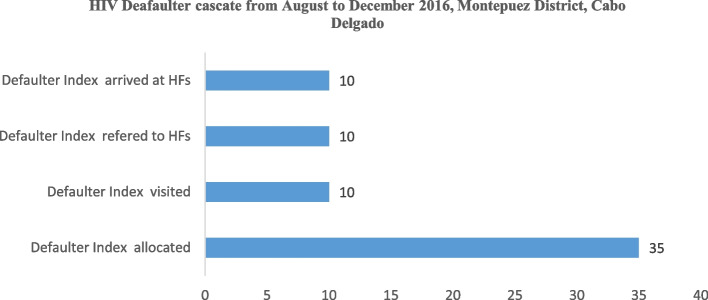
Infomóvel tracing first pilot phase results from August to December 2016, Montepuez District, Cabo Delgado

Evaluation of the first pilot phase, which was conducted in a single healthcare institution in the Montepuez district of Cabo Delgado Province, reveals respectable levels of patient adherence and retention in the care and treatment provided by the healthcare facilities to the community. From August to December 2016, the following information was gathered from Infomóvel in Montepuez: 145 patients who were visited out of the 244 allocated index patients, 368 cohabiting patients were recorded as the predicted index patient. Eight of the 126 cohabiting individuals who underwent testing for HIV were given referrals to the Rural Hospital of Montepuez, and five additional individuals were recorded as arriving at this medical facility. These data show that 59% of index patients were contacted by APE for health education, which resulted in 34% of tested cohabiting patient and 5/8 of HIV positive cohabiting that were enrolled at the health facility (Fig. 6). There were 35 absent or defaulter patients, however 10 were located, 10 referred to the Rural Hospital, and 10 arrived. Although 10/35 of defaulter were located, 10 of them when located returned to care and treatment at the Rural Hospital of Montepuez (Fig. 7). The platform generates dynamic reports and cascades that make it possible to evaluate and interpret data in order to assist Ariel in choosing the most important programs to implement for prevention and treatment.

The second pilot phase was used to evaluate the utility, with default and freshly begun ART patients, TB and PMTCT mother-baby pair to follow-up, and nutrition modules that were included.

**Fig. 8:**
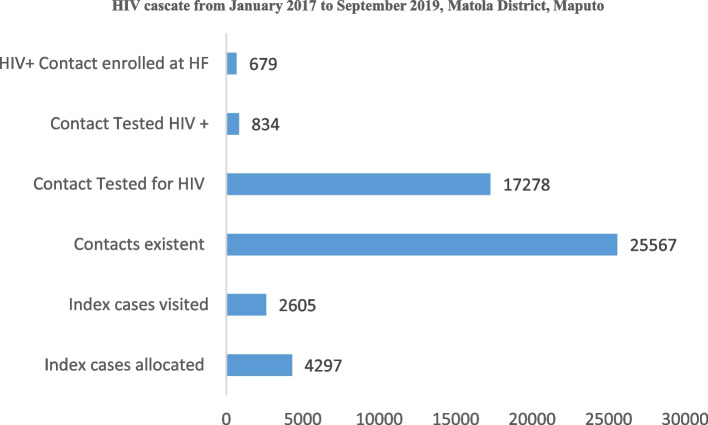
Infomóvel second phase pilot results, from January 2017 to September 2019, Matola District, Maputo

In total, the pilot registered and assigned 4,297 index patients, most of which were adults (15 years or more). The platform located 2,605 (61%), from those 17,278 (68%) that were recorded as cohabiting index patient of 25,567 total cohabitants expected. About 17,278 cohabitants were tested, of which 834 (5%) were diagnosed with HIV, and referred to health facilities in Matola district, and 679 (81%) were recorded as arrivals on this health facility and were enrolled on treatment (Fig. 8).

**Fig. 9:**
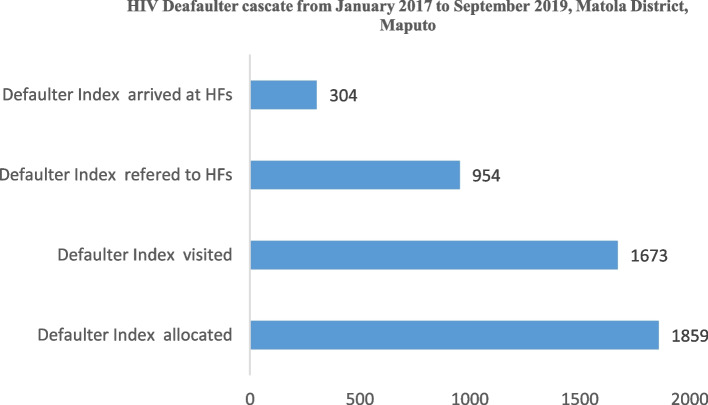
Infomóvel second pilot phase results, from January 2017 to September 2019, Matola District, Maputo

In total, the pilot registered and assigned 1,859 defaulter patients. The platform located 1,673 (90%) of the index patients (patients in ART default), 954 were referred and ensured that 304 (32%) returned to the health facility, and more than half resumed ART treatment (Fig. 9).

Other notable successes from anecdotal observations during the pilot include improved patient flow within the health facility, decreased number of patients actively in default status, and improved communication and coordination between the health facility and communities. The main obstacles were worries about the workload of CHWs and their capacity to test every qualified cohabitant. It has been anecdotally stated that the mobile application is user-friendly and has a well-designed training. However, the pilot did not evaluate how the CHWs utilized Infomóvel in the field and their impression of the utility and influence on counseling and active search for index patients. It will be completed at a later time during the evaluation of Infomóvel's implementation.

## Discussion

The development of a new mHealth platform was promoted to enable an increase in the identification of HIV positive individuals, increase in linkage to HIV services, adherence to treatment and retention in care. Referrals of HIV positive cases found in the community to medical institutions can help to lower rates of antiretroviral treatment abandonment and enhance the detection of HIV patients in the community, which will hasten the accomplishment of the global first and second ninety-five goals [4]. The pilot's outcomes, which depict how patients are managed in the HIV program from medical facilities to the community and vice versa, were overwhelmingly favorable and showed a definite improvement in access to HIV care. The use of the DSRM methodology allowed the validation of the pilot results to contribute to strengthen the management of patients living with HIV, aspects that will be discussed in this section.

### Pilot research findings

In general, we can state that the pilot's outcomes enabled the identification of the key players, the structural environment, and the strengths and weaknesses of this HIV mHealth effort in Mozambique in order to foster synergies during the implementation and scaling up phases. Other contexts also employed the same methodology [18].

Before the Infomóvel platform was introduced, it was necessary to start with a paper-based index case program to familiarize the case manager at the health facility and the CHWs in the community with the methodology because it isn't frequently used at health facilities and communities. This was necessary due to the structural context of Mozambique. After 6 months of paper-based approach this solution-Infomóvel platform, implemented aligned with the context.

The main challenge in the testing phase is concerned with the quality of information extracted from the Patients Tracking System such as the address and contacts of patients which are the basis for the success of the program. Another challenge was the educational level and the ability to carry out HIV testing and handling of smartphones by the APE, as most of them only had grade eight, and this is a point that was taken into consideration in the pilot phase. Finally, the connection between the Infomóvel and the Patient Tracking Systems should be automated to ensure the effective and efficient patient follow-up.

An essential component of the Infomóvel process is the educational background of both case managers and CHWs. Both must be literate in order to participate in the two types of training—one focused on health knowledge and the other on technological issues in order to handle the three types of visits already mentioned, as well as patient home visits. The application records the geographical coordinates, followed by all the data that the CHWs collect, such as demographics, diagnosis of various symptoms, estimation of ART adherence, record of the cohabitants and their results of rapid tests, counseling, health education and patients’ referral to health facility. Although educational background was a factor in the hiring process, it was easy to find all case managers and community health workers locally, which helped in the implementation because they are familiar with the communities and health catchment regions as well as the GAAC members [34].

The 22 APEs received training in the first pilot phase on a variety of clinical and psychosocial topics, including home visits, counseling, disclosure and adherence, HIV testing, testing quality, and, ultimately, practical issues linked to mobile phone application usage. A case manager, CHWs, and an APE supervisor were also taught on the platform. The case manager's duties include preparing patient data from a patient health facility tracking system for the Infomóvel platform, allocating patients to CHWs based on domicile, accepting patients referred by communities, and monitoring them for clinical services.

The mobile phone offers a user-friendly tool for CHWs and APE, is simple to use and quick to process information, making it a solid and very trustworthy instrument for community-based programs for counseling and testing. It makes the process of testing, registering, advising, and referring patients easier. Many patients can receive warning SMSs, recommendations to return to HF, or assistance in keeping them in care and treatment thanks to automatic alert mechanisms. The Montepuez district government and local leaders participated as a beneficiary of the approach, helping to build community interaction and supporting advocacy for community engagement throughout the platform construction process.

Other notable successes from anecdotal observations during the second pilot phase include improved patient flow within the health facility, decreased number of patients actively in default status, and improved communication and coordination between the health facility and communities.

Using community-based health professionals to reach, test, and refer HIV and TB patients has shown overwhelmingly favorable results in literature and experience outside of Mozambique [7–12]. Similar to what happened in several studies, Infomóvel demonstrated the positive impacts of using mobile technology to strengthen community-level management of suspected HIV, and to reinforce links between CHWs and district health facilities and improve counseling and testing, linkage to care, and treatment adherence [14–16], with a counseling and testing rate of 68% of contacts of index cases, from all HIV positives identified at communities; the community linkage rate to the health facilities was 81%. The pilot of Infomóvel demonstrated feasibility for increasing the efficiency to reach patients who had defaulted on ART, with rates of 90% of lost-to-follow up patients identified in the communities (Fig. 7).

Active case finding techniques for both HIV and TB within communities thought to be at high risk have been supported, similar to the index case methods used by Infomóvel [35]. In Cambodia, CHWs conducted active door-to-door screening and sputum sample collection for TB, sending test results and referrals via SMS to the health facility [36]. This active testing and referral system was well received by patients and health facilities, however the treatment completion rates from this screening method (81%) were lower than passive case finding programs in same setting (90%), but still reasonable, and likely caused by the low uptake of treatment by new patients who were not willing or able to seek treatment at health facility [36]. Community-based TB testing also showed positive results in Peru, the TB prevalence detected through combined active and passive case finding among 1,094 household contacts was 0.91% (914 per 100,000), much higher than with passive case finding alone (0.18%; 183 per 100,000; *p* = 0.02) [37]. In multiple settings, active case finding (home visits by CHWs & home testing) was a key element in increasing identification of people with active TB, increasing the yield of new patients by up to 26% [38]. Due to community-based patient tracing and counseling, additional regional evidence suggests that community-based mHealth approaches improved social support, privacy (contrary to expectations), and reduced stigma. However, no changes in rates of virologic failure, adherence, mortality, or retention were observed, with the exception of virologic failure in the long term (> 96 weeks) [34–36]. Similar to this, CHWs in Kenya replaced monthly clinic visits for HIV + patients with home visits utilizing PDAs to track adherence, check for side effects of therapy, and refer or follow up as appropriate. The results of this program's evaluation demonstrated that CHWs can successfully replace clinic appointments without substantially reducing clinical outcomes [39].

Given that 80% of the total number of new ART patients offered the method and subsequently registered in patients tracking system (ePTS or OpenMRS) accepted the home visit, the initiative’s reception by HIV + patients was favorable.

The pilot of Infomóvel also demonstrated that the CHWs and case managers are a more effective way to actively conduct patients search and test eligible contacts. This approach can be considered for the community follow-up of chronic patients, not only for HIV, but other chronic diseases if the patients are stable such as hypertension and diabetes.

In addition to allowing its use even in regions with limited access to the Internet network, Infomóvel records the offline geographic coordinates of the home visited, along with all the programmatic data that the activist collects, and the demographic data. This creates an opportunity for this information to be used to map HIV Hotspot areas, which can assist health managers in making decisions for better policy formulation and program implementation. On the other hand, the infomóvel platform also registers all CHWs activity on the platform, which facilitates the monitoring of their performance, CHWs supervision and identified training needs.

### Implications for research

In this work, the authors test the DSRM as a method to develop new digital health services, which has already been demonstrated in other studies [40]. However, the use of the DSRM methodology in development of technological platforms for HIV services in community is still a novelty. The Infomóvel platform proposal improves access to HIV testing services as well as follow-up of HIV-positive patients on antiretroviral treatment in the communities, reducing rates of treatment abandonment. The DSRM methodology has made it possible to identify the right CHWs to study the contributions of the Infomóvel platform proposal.

Considering the DSRM methodology, the Infomóvel platform complied with four principles of Österle's for investigating design-oriented information systems: abstraction, originality, justification and benefits [41].

Abstraction: Infomóvel can be used by any CHWs, who can read and write and who has been trained in HIV subjects as HIV counseling/testing and community follow-up of HIV positive patients.

Originality: Infomóvel was designed by a multidisciplinary team including physicians, psychologists, nurses, statisticians and computer engineers, with input from CHWs and HIV patients, contributing to the creation of a system oriented to their real needs. For the first time, a community health system was implemented in Mozambique to monitor HIV-positive patients in the community.

Justification: UNAIDS data show that HIV/AIDS affects about 37.9 million individuals in the world, of this 67% are in Sub-Saharan Africa being Mozambique the fourth country with the smallest decrease in the number of new infections [4]. The main challenge of the HIV program is the retention of patients on anti-retroviral treatment [42]. Infomóvel is an mHealth service that helps to improve access to community HIV testing as well as in the follow-up of HIV-positive patients on antiretroviral treatment in the community in order to prevent lost follow and abandoned to treatment.

Benefits: The preventive follow-up of HIV-positive patients on antiretroviral treatment in the community reduces lost follow up and abandon and helps to identify people living with HIV through index case testing, contributing to improve access to HIV services.

### Implications for community services practice

The degree of education and reading and writing skills of CHWs have a significant impact on their capacity to deliver community services utilizing a mHealth platform. On the other hand, since the CHWs were residents of the neighborhood, there was more opportunity for patient interaction. This is an important point to make since building trustworthy connections with patients is essential to providing community services, especially in the case of Mozambique. The ability of CHWs to receive training in both programmatic and technological areas to control the flow of various Infomóvel processes using smartphones has a significant impact on the quality of the services they deliver. This viewpoint suggests a new way of operating through a standardized approach in the programmatic as well as in the use of technology to document services provided and their monitoring by CHWs in the delivery of HIV community services. The Infomóvel allows an active management of HIV-positive patients at the community level that permit their follow-up and preventive support,and reduce the workload on the CHWs since the platform allow the reception of information from health facilities about patients to be visited without they having to move the health facilities. The Infomóvel platform makes it possible for CHWs to have real-time access to patient information, who they must accompany in the community, and, when they are located, to share all the information about services provided, the reasons for lost follow-up, as well as information regarding the HIV testing of their family members, if there is internet access. This strengthens the connection between the HIV services provided by the health facilities and those provided in the communities. The infomóvel allows both the case manager in the health facility and the CHWs in the community to use the platform in a reciprocal way, sharing information which allows data validation and reducing the visit of CHWs to the health facility which reduces overload and transportation costs.

The infomóvel is an innovation since it replaced the manual, paper-based environment in which community HIV services are offered with an entirely digital, mHealth model. The use of Infomóvel has greatly decreased the burden for case managers and CHWs, enhanced the quality of services through the development of standard operating procedures, and enhanced the program's impact through increased follow-up of patients who default in communities. The ultimate goal of using technology as an innovation is to improve the quality of life for patients living with HIV.

## Conclusion

The use of the DSRM has helped to implement a community-based HIV service delivery involving health facility and community actors, including patients. Through this methodology it was possible to create standard procedures that allowed the reorganization of HIV community services and engaging the CHWs. Thus, Infomóvel offers procedures that allow standardized follow-up of patients in the communities, as well as through index case to identify more people living with HIV.

The platform’s functionality and quality are crucial because, even though it exists, CHWs must be able to manage it. Additionally, there needs to be a trustworthy relationship between CHWs and patients so that patients feel at ease, receive home visits, and develop a growing sense of responsibility for disease management.

In this study, it was feasible to confirm that the use of mHealth technology helped to improve both the follow-up of HIV-positive individuals receiving antiretroviral treatment as well as the identification of people living with HIV in the communities using the index case technique. These two aspects contributed to an improved access to services and retention of patients on ART, contributing to the improvement of UNISIDA's global goals for the first and second ninety. Based on the Infomóvel pilot study's findings, it is clear that the deployment of mHealth strategies in Mozambique is supported by a growing body of research and that these strategies may be scaled up nationally. This experience shows that mHealth initiatives can be better exploited in Mozambique, and elsewhere, to manage patients with chronic diseases such as hypertension and others, as long as the patients are stable. After this pilot study, however, more research should be done in order to estimate the funding required to use the strategy on a larger scale.

### Aviability and requirements

Project name: Infomóvel.

Project home page https://www.commcarehq.org/a/infomovel-pepfar

Operanting system: android and web-based (support all browser).

Programming language:is based in commcare.

Other requirements: android 5.1 or later.

License:GNU GPL.

Any restrictions: license needed.

## Data Availability

The platform is hosted on CommCare, an open-source system located at https://www.commcarehq.org, whose flows can be consulted freely. All the documentation related to the creation of the platform is archived and available for consultation whenever necessary. Data hosted and archived on the platform is also available for assessment of access to HIV services, follow-up and evaluation of patients adherence and retention.

## Abbreviations

AIDS: Acquired immune deficiency syndrome
APE: *Agente polivalente elementar*
ART: Antiretroviral treatment
CASG: Community adherence and support groups
CHWs: Community health workers
DSRM: Design science research methodology
ePTS: Patient tracking system
GAACs: *Grupos de Apoio a Adesão Comunitária*
HF: Health facility
HIV: Human immunodeficiency virus (HIV)
LMIC: Low and middle-income countries
mHealth: Mobile health
MoH: Ministry of Health
PLWHIV: People living with HIV
PMTCT: Prevention of mother to child transmission
TB: Tuberculosis
UNAIDS: United Nations programme on HIV/AIDS
RCT: Randomised controled trial
GDP: Good documentation practice
